# Short-Term Exercise Training Improves Insulin Sensitivity but Does Not Inhibit Inflammatory Pathways in Immune Cells from Insulin-Resistant Subjects

**DOI:** 10.1155/2013/107805

**Published:** 2013-03-13

**Authors:** Sara M. Reyna, Puntip Tantiwong, Eugenio Cersosimo, Ralph A. DeFronzo, Apiradee Sriwijitkamol, Nicolas Musi

**Affiliations:** ^1^Medical Research Division, Regional Academic Health Center, 1214 W. Schunior Street, Edinburg, TX 78541, USA; ^2^Diabetes Division, University of Texas Health Science Center at San Antonio, 7703 Floyd Curl Drive, San Antonio, TX 78229, USA; ^3^Texas Diabetes Institute, 701 S. Zarzamora, San Antonio, TX 78207, USA; ^4^Geriatric, Research, Education, and Clinical Center, Audie L. Murphy VA Hospital, 7400 Merton Minter Boulevard, San Antonio, TX 78229, USA

## Abstract

*Background*. Exercise has an anti-inflammatory effect against, and immune cells play critical roles in the development, of insulin resistance and atherosclerotic vascular disease (AVD). Thus, the goal of this study was to determine whether exercise improves insulin sensitivity in insulin-resistant subjects by downregulating proinflammatory signaling in immune cells. *Methods*. Seventeen lean, 8 obese nondiabetic, and 11 obese type 2 diabetic individuals underwent an aerobic exercise program for 15 days and an insulin clamp before and after exercise. Peripheral mononuclear cells (PMNC) were obtained for determination of Toll-like receptor (TLR) 2 and 4 protein content and mitogen-activated protein kinase phosphorylation. *Results*. Compared with that in lean individuals, TLR4 protein content was increased by 4.2-fold in diabetic subjects. This increase in TLR4 content was accompanied by a 3.0-fold increase in extracellular signal-regulated kinase (ERK) phosphorylation. Exercise improved insulin sensitivity in the lean, obese, and type 2 diabetes groups. However, exercise did not affect TLR content or ERK phosphorylation. *Conclusions*. TLR4 content and ERK phosphorylation are increased in PMNC of type 2 diabetic individuals. While exercise improves insulin sensitivity, this effect is not related to changes in TLR2/TLR4 content or ERK phosphorylation in PMNC of type 2 diabetic individuals.

## 1. Introduction

 Regular exercise is fundamental for the prevention and management of insulin-resistant disorders such as type 2 diabetes mellitus (T2DM) and AVD. While exercise affects numerous organ systems, there is increasing evidence that acute and chronic exercises have important immunomodulatory properties which may contribute to the beneficial effects of exercise on metabolism and cardiovascular health [1]. For example, aerobic exercise improves insulin sensitivity [2] and enhances endothelial function [3]. In addition, aerobic exercise can alter the number and the function of immune cells in innate and adaptive immunities [4–6] and reduce the levels of proinflammatory markers [7].

 Numerous reports have demonstrated that immune cells play a critical role in the development of insulin resistance. Hevener et al. showed that deletion of peroxisome proliferator-activated receptor gamma (PPAR*γ*) in monocytes results in glucose intolerance and decreased insulin signaling in the skeletal muscle, liver, and adipose tissue of mice [8]. Another study demonstrated that regulatory CD4^+^ T cells expressing the transcription factor Foxp3 (T_reg_ cells) are predominately found in the abdominal fat of normal mice but are reduced in the abdominal fat in mouse models of insulin resistance [9]. This study also showed that interleukin-10 (IL-10), produced by these Foxp3 T_reg_ cells, inhibited tumor necrosis factor-alpha (TNF*α*)-induced expression of proinflammatory mediators and insulin resistance in 3T3-L1 adipocytes. Taken together, these findings suggest that immune cells have the potential to affect whole body glucose homeostasis and insulin action.

 In addition to their role in insulin resistance, immune cells are also involved in the development of atherosclerosis [10]. In T2DM patients, atherosclerosis is systemic, rather than localized to the heart, and also affects the microvasculature of the limbs, kidneys, eyes, and other tissues [11]. Monocytes play crucial roles in the early events that lead to this systemic atherosclerosis [12]. Yet, the molecular mechanisms that promote accelerated atherosclerosis in type 2 diabetic subjects are not fully understood. Accumulating evidence implicates Toll-like receptor (TLR) 2 and 4 in the pathogenesis of atherosclerosis and insulin resistance [13–16]. For example, monocytes from type 2 diabetic subjects have elevated surface protein content of TLR2 and TLR4 [17]. Moreover, deletion of TLR2 or TLR4 in rodents attenuates atherosclerosis and insulin resistance [14, 15, 18, 19].

 In view that immune cells have a critical role in the pathophysiology of insulin resistance and AVD, in this study, we examined the molecular mechanism underlying the anti-inflammatory effect of aerobic exercise on immune cells of type 2 diabetic individuals. Although some studies have shown that either aerobic or resistance exercise induces a decrease in TLR4 expression in monocytes from healthy normal subjects [4, 20, 21], it is unclear whether aerobic exercise can modulate proinflammatory signaling pathways in immune cells of insulin-resistant subjects. It is well documented that aerobic exercise can improve insulin sensitivity in type 2 diabetic patients [2]. However, no study has described whether the improvement of insulin sensitivity is due to a decrease in proinflammatory signaling pathways in immune cells of type 2 diabetic subjects. We focused our analysis on PMNC because of the considerable evidence indicating that these inflammatory cells infiltrate insulin-sensitive tissues (skeletal muscle, adipocytes, and liver) where they promote insulin resistance [22–24] and are also involved in the pathophysiology of AVD [10]. Therefore, we hypothesized that aerobic exercise downregulates proinflammatory signaling pathways in PMNC of insulin-resistant individuals.

## 2. Materials and Methods

### 2.1. Study Participants

We studied 17 lean, 8 obese nondiabetic, and 11 obese type 2 diabetic participants. Five T2DM subjects were diet-treated. Three T2DM subjects were newly diagnosed, and one T2DM subject was diagnosed with diabetes 2 months prior to the study. Two T2DM subjects took a sulfonylurea, which was stopped 2 days before any study procedure. All participants were sedentary (zero or one exercise bout per week) and had stable body weight (±1 kg) for three months before the study. Each participant underwent a medical history, physical examination, and a 75 g oral glucose tolerance test (OGTT). Lean and obese participants did not have a family history of type 2 diabetes and were normal glucose tolerant. No participant was taking any medication known to affect glucose metabolism (other than sulfonylureas). The study was approved by the Institutional Review Board of the University of Texas Health Science Center at San Antonio, and all participants gave written consent.

### 2.2. Oral Glucose Tolerance Test (OGTT)

Plasma glucose, insulin, and nonesterified fatty acids (NEFA) were measured 30, 15, and 0 minutes before and every 15 min for 2 h after the ingestion of 75 g of glucose.

### 2.3. *V*O_2peak_ Testing

Within 3–7 days after the OGTT, *V*O_2peak_ was determined using a cycle ergometer and a Metabolic Measurement System (Sensormedics, Savi Park, CA, USA) as previously described [25].

### 2.4. Insulin Clamp

Within 1-2 weeks after the baseline *V*O_2peak_ measurement, participants returned to the Clinical Research Center at 07:00 AM to undergo a 180 min euglycemic-hyperinsulinemic (160 mU · m^2^ · min⁡^−1^) clamp study as previously described [26].

### 2.5. Exercise Training Protocol

Within one week of the insulin clamp, participants undertook a supervised exercise program of cycle ergometer exercise for 40 min per day, for 15 consecutive days. The 40 min of exercise consisted of 4 identical 10 min periods comprised of 8 min of exercise at 70%  *V*O_2peak_ followed by 2 min at 90% of *V*O_2peak_. Each of these 10 min sets was followed by 2 min of complete rest. We employed this program because, during pilot experiments, we observed increases in *V*O_2peak_ of ~20%. *V*O_2peak_ testing was done on the last day of the training protocol. The insulin clamp was repeated 36 h after the last exercise session.

### 2.6. Mononuclear Cell Isolation

Blood samples were collected immediately before and at the end of the pre- and postexercise insulin clamps for isolation of PMNC. PMNC were isolated using a Histopaque-1077 (Sigma, St. Louis, MO, USA) density gradient, followed by centrifugation at 800 ×g for 30 min at room temperature. The PMNC from the interface were collected and washed three times with PBS. 

### 2.7. Western Blotting

Mononuclear cell (1 × 10^7^cells) samples were lysed in ice-cold lysis buffer (pH 7.4), and Western blot analyses were performed as previously described [13]. After blocking with bovine serum albumin, the membranes were incubated overnight with primary antibody against TLR4 (Santa Cruz Biotechnology, Santa Cruz, CA, USA) and TLR2 (eBioscience, San Diego, CA, USA), which are cell surface receptors that signal through ERK and c-Jun amino-terminal kinase (JNK). We also performed Western blotting for phosphorylated ERK (Invitrogen, Carlsbad, CA, USA), ERK (Cell Signaling, Danvers, MA, USA), phosphorylated JNK (Cell Signaling, Beverly, MA, USA), and JNK (Cell Signaling, Beverly, MA, USA). Bound primary antibodies were detected with a secondary antibody (anti-rabbit immunoglobulin-horseradish peroxidase-linked antibody) using enhanced chemiluminescence reagents. Bands were quantitated with ImageQuant (GE Healthcare, Piscataway, NJ, USA). 

### 2.8. NF*κ*B Activity

NF*κ*B p65 binding was measured using an ELISA kit (Active Motif, Carlsbad, CA, USA), as previously described [13]. 

### 2.9. Laboratory Analysis

Plasma insulin (Diagnostic Products, Los Angeles, CA, USA) was measured by radioimmunoassay. Glucose was measured by the glucose oxidase method using a Beckman Glucose Oxidase Analyzer, and hemoglobin A1C (HbA_1c_) was measured using a DCA2000 analyzer (Bayer, Tarrytown, NY, USA). Plasma NEFA concentrations were determined using a colorimetric method (Wako, Richmond, VA, USA). High-sensitivity-C-reactive protein (hs-CRP) was measured by ELISA (ALPCO Diagnostics, Salem, NH, USA). Endothelin-1 and soluble intercellular adhesion molecule-1 (sICAM-1) plasma levels were measured by ELISA (R&D Systems, Minneapolis, MN, USA).

### 2.10. Statistical Analysis

Data are expressed as mean ± SE. Baseline comparisons between groups were done using one-way ANOVA, and the effect of exercise training and insulin was analyzed using ANOVA with repeated measures followed by the Tukey test. SigmaStat version 3.5 (Systat software, San Jose, CA, USA) was used for statistical analysis. 

## 3. Results

### 3.1. Study Participants

Table 1 shows the characteristics of study participants before and after the 15-day aerobic exercise training program. Compared with the lean group, the obese and type 2 diabetic subjects had higher body mass index (BMI) and body weight. There was a small but significant difference in age between groups. Plasma HbA_1c_ and NEFA concentrations were elevated in the diabetic patients. The obese group tended to have elevated plasma NEFA concentrations compared with the lean group (*P* = 0.056). Type 2 diabetic individuals had higher fasting glucose levels. Both diabetic and obese subjects had higher fasting insulin levels when compared with the lean group. The type 2 diabetic patients had higher levels of the inflammatory and endothelial markers hs-CRP, endothelin-1, and sICAM-1. Diabetic and obese individuals were insulin resistant compared to the lean controls, based on the lower total glucose disposal during the insulin clamp. 

 After the exercise training, the insulin-resistant individuals (obese and T2DM groups) did not show a significant decrease in BMI or weight. Similarly, there was no change in HbA_1c_, NEFA, glucose, and insulin plasma levels in the obese and diabetic groups. Exercise also did not decrease the plasma levels of hs-CRP, endothelin-1, and sICAM-1. 

### 3.2. Effect of Exercise Training on *V*O_2peak_ and Insulin Sensitivity

After exercise, *V*O_2peak_ increased by 26% (*P* < 0.05), 14% (*P* = 0.08), and 8% (*P* < 0.05) in lean, obese, and diabetic individuals, respectively. The baseline M levels were 10.9 ± 0.7, 7.8 ± 0.8, and 4.2 ± 0.6 mg/kg·min in lean, obese, and type 2 diabetic individuals, respectively. After exercise, insulin sensitivity (*M* values) increased by 11% (*P* < 0.05) in lean, 15% (*P* < 0.05) in obese, and 32% (*P* < 0.05) in type 2 diabetes groups.

### 3.3. TLR Protein Content

**Figure 1:**
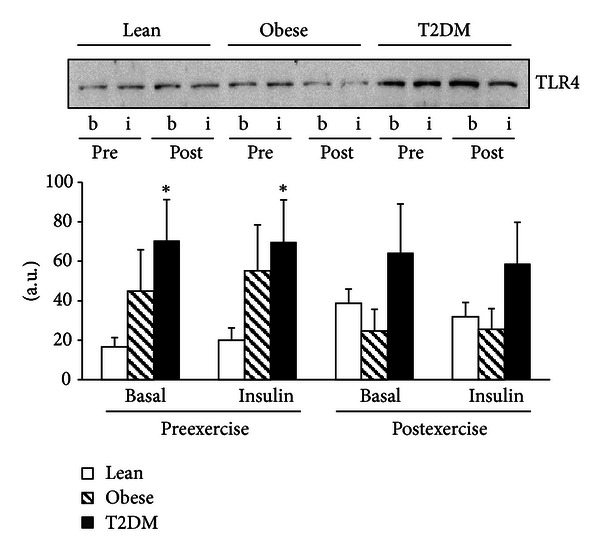
TLR4 protein content. TLR4 was measured in PMNC from 17 lean, 8 obese, and 11 T2DM individuals at the basal state (b) and at the end of the insulin clamp (i) before (pre) and after (post) exercise training. Data are means ± SE. Blots are shown for one lean, one obese, and one T2DM individual. *P < 0.05 versus lean at basal preexercise training. a.u.: arbitrary units.

**Figure 2:**
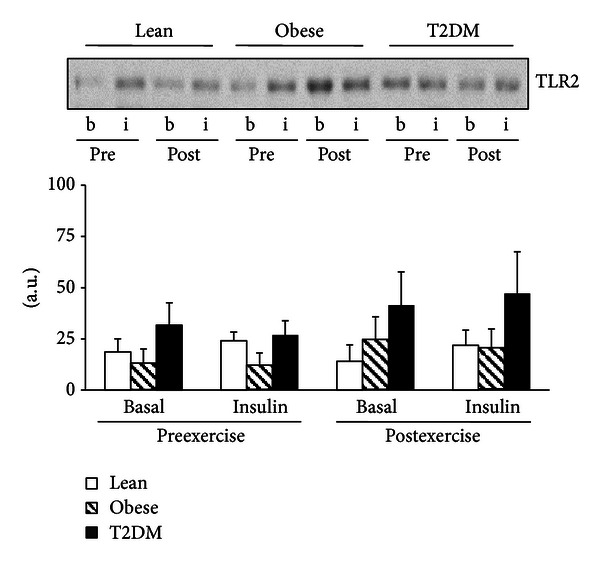
TLR2 protein content. TLR2 was measured in PMNC from 17 lean, 8 obese, and 11 T2DM individuals at the basal state (b) and at the end of the insulin clamp (i) before (pre) and after (post) exercise training. Data are means ± SE. Blots are shown for one lean, one obese, and one T2DM individual. a.u.: arbitrary units.

Compared with lean participants, baseline TLR4 protein content was significantly increased in the PMNC of diabetic subjects by 4.2-fold (*P* < 0.05) (Figure 1). In obese individuals, TLR4 also tended to be elevated by 2.7-fold (*P* = 0.07) compared to that of the individuals in the lean group (Figure 1). Exercise training and insulin infusion did not affect TLR4 levels. In contrast to TLR4, the protein content of TLR2 was not different in lean, obese, and diabetic subjects (Figure 2). Neither exercise training nor insulin infusion changed the levels of TLR2 in PMNC.

### 3.4. ERK Phosphorylation

**Figure 3:**
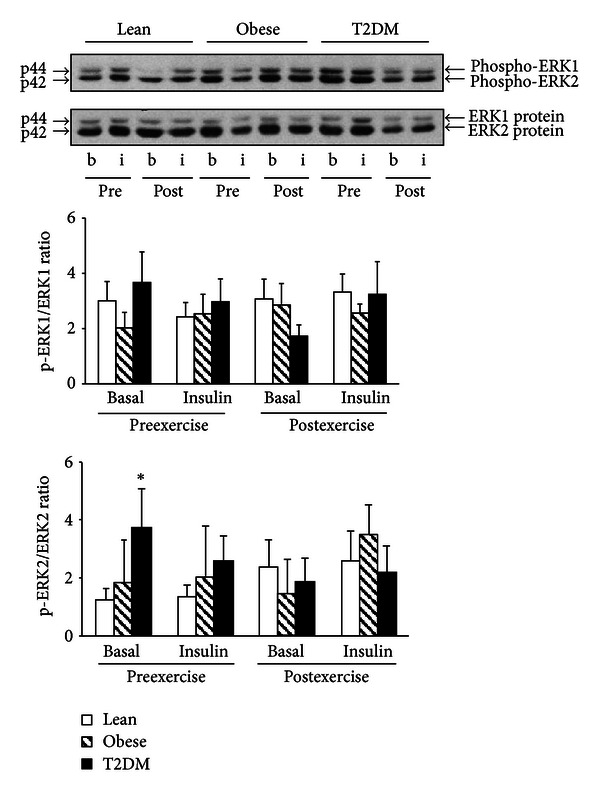
ERK phosphorylation and protein content. Phosphorylation of ERK 1 and 2 and ERK 1 and 2 protein was measured in PMNC from 17 lean, 8 obese, and 11 T2DM individuals at the basal state (b) and at the end of the insulin clamp (i) before (pre) and after (post) exercise training. Data are means ± SE. Blots are shown for one lean, one obese, and one T2DM individual. *P < 0.05 versus lean at basal preexercise training. a.u.: arbitrary units.

As shown in Figure 3, baseline ERK phosphorylation was significantly elevated in the diabetic subjects. The increase in ERK phosphorylation was mainly observed with ERK2 (3.0-fold versus the lean group; *P* < 0.05). ERK2 phosphorylation tended to be decreased in type 2 diabetic subjects after exercise (*P* = NS).

 ERK phosphorylation was not significantly affected by hyperinsulinemia during the clamp. We performed these measurements just before the end of the 180 min clamp, based on previous studies that showed that insulin activation of ERK occurs as late as 100–240 min during a clamp [27, 28]. It is possible, however, that insulin could have activated ERK if measurements had been done at earlier time points. 

### 3.5. JNK Phosphorylation

**Figure 4:**
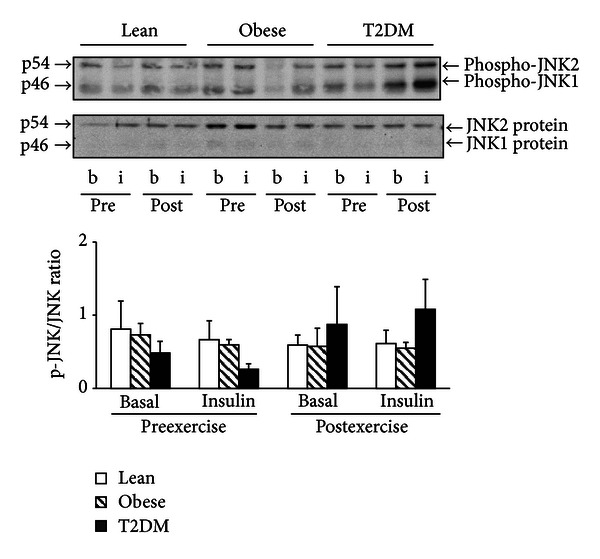
JNK phosphorylation and protein content. Phosphorylation of JNK and JNK protein was measured in PMNC from 17 lean, 8 obese, and 11 T2DM individuals at the basal state (b) and at the end of the insulin clamp (i) before (pre) and after (post) exercise training. Data are means ± SE. Blots are shown for one lean, one obese, and one T2DM individual. a.u.: arbitrary units.

There were no significant differences in JNK phosphorylation (Figure 4) between groups. JNK phosphorylation was not affected by insulin infusion or exercise (Figure 4).

### 3.6. NF*κ*B p65 Binding

**Figure 5:**
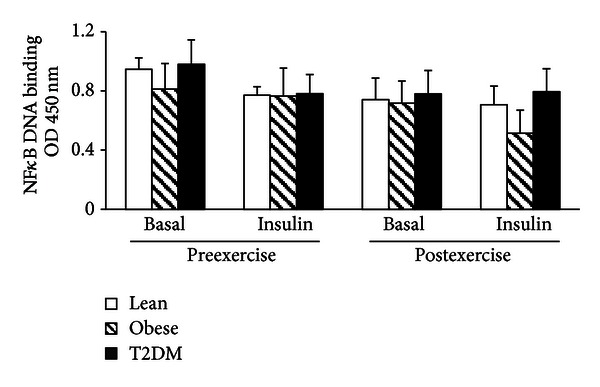
NF*κ*B activity. NF*κ*B DNA binding was measured in PMNC from 17 lean, 8 obese, and 11 T2DM individuals at the basal state (b) and at the end of the insulin clamp (i) before (pre) and after (post) exercise training. Data are means ± SE.

NF*κ*B p65 DNA binding was not different between lean, obese, and type 2 diabetic participants, and neither exercise nor insulin affected NF*κ*B DNA binding (Figure 5).

## 4. Discussion

 There is evidence suggesting that exercise has an anti-inflammatory effect [7]. Exercise training studies and cross-sectional comparisons between physically active and inactive individuals have shown a reduction in the inflammatory response to LPS and lower TLR4 cell surface expression in monocytes from physically active subjects [20, 21]. Because immune cells play a role in the regulation of glucose metabolism [8, 29], we examined whether exercise improves insulin sensitivity by inducing modifications in the pro-inflammatory signaling pathways of the PMNC of insulin-resistant subjects. We anticipated that short-term training would improve insulin sensitivity in insulin-resistant individuals and that this would then be associated with a decrease in TLR4 protein content and ERK signaling in PMNC. Yet, acute aerobic exercise, which improved insulin sensitivity, did not affect TLR4 protein content, although it tended to attenuate ERK2 phosphorylation. It is possible, however, that the 15-day intervention was not sufficient to significantly alter TLR4 protein content and ERK phosphorylation. On the other hand, others have reported that a single session of cycle exercise (1.5–2 h long) is sufficient to decrease TLR4 cell surface expression in monocytes from healthy individuals [30], although TLR-downstream signaling (ERK) was not examined in that study. The apparently discrepant results between the present and previous studies may be related to differences in the duration of training, the type of training being performed (aerobic, resistance, or combination), the intensity of the training (moderate versus high), and/or the population examined. Timmerman et al. tested the effect of combined aerobic and resistance training on monocyte subpopulations and found that TLR4 expression was reduced in “proinflammatory” (CD14^+^16^+^) but not in “classical” (CD14^+^) monocytes [31]. Proinflammatory monocytes adhere more avidly to activated endothelial cells and are precursors to the CD16^+^ macrophages which are distributed throughout atherosclerotic lesions [32]. We cannot rule out that the exercise intervention employed in the present study could have led to changes in TLR4 protein content and ERK signaling in specific monocyte subpopulations. 

 In this study, we found that insulin-resistant individuals have elevated TLR4 protein content in PMNC. Our findings are in agreement with previous studies where TLR levels were found to be increased in inflammatory cells from type 1 and type 2 diabetic subjects, although, in contrast to the present study, subjects had increased levels of both TLR2 and TLR4 [17, 33]. The physiologic relevance of the increased TLR4 protein content is underscored by studies in which TLR4 was ablated specifically in hematopoietic cells from mice, an intervention which reduced inflammatory markers in liver and adipose tissue and ameliorated high-fat diet-induced insulin resistance [34]. Moreover, our results also demonstrate that the upregulation in TLR4 protein levels is accompanied by an increase in ERK phosphorylation, especially ERK2. Studies conducted in* ob/ob-Erk1* ^−/−^ double knockout mice showed that ERK deletion improves insulin sensitivity in skeletal muscle and reduced liver fat content [35]. Defining the role of ERK2 on insulin resistance has been challenging because its genetic ablation (globally) leads to embryonic death [36]. Future studies of tissue-specific ERK2 ablation will help to clarify its role in the pathogenesis of insulin resistance and type 2 diabetes.

 AVD is the major cause of mortality in subjects with type 2 diabetes and insulin resistance [37]. Atherosclerosis occurs earlier in the natural course of diabetes and is more severe and generalized than in nondiabetic patients [37]. To test whether TLR4 has a direct role in the pathogenesis of atherosclerosis, Michelsen et al. crossed apolipoprotein E (ApoE) deficient mice, which are prone to develop atherosclerotic lesions, with mice that are null for TLR4 [19]. This group demonstrated that mice deficient in both TLR4 and ApoE have a significant reduction in the size of the atherosclerotic lesions, as well as a lower number of macrophages infiltrating these lesions, compared with mice deficient with ApoE only [19]. More recently, it was reported that hematopoietic cell ablation of TLR4 reduces arterial wall macrophage infiltration and atherosclerotic lesion areas [38]. ERK also has a crucial role in the early steps in the pathogenesis of atherosclerosis because activation of ERK leads to increased monocyte proliferation, migration, and differentiation into macrophages [39] and mediates monocyte chemotactic protein-1-induced monocyte adhesion [40]. Indeed, (monocyte-derived) dendritic cells from patients with acute coronary syndrome have increased ERK phosphorylation [41]. In addition, a recent study demonstrated that hypertensive patients have increased ERK phosphorylation in isolated white blood cells compared to healthy normotensive individuals, suggesting that ERK activation in leukocytes may serve as a biomarker for hypertension [42]. Collectively, the results from these studies and our present findings strongly suggest that enhanced signaling through TLR4 and ERK, in inflammatory cells (monocytes/macrophages), plays an important role in the pathogenesis of atherosclerosis.

 TLR4 protein content and ERK signaling were elevated in the type 2 diabetes group, which were slightly but significantly older than the lean and obese groups. Based on previous reports from human and animal studies [21, 43, 44], we believe that the increase in TLR4 protein content and ERK signaling is not due to the small difference in age. In fact, Stewart et al. compared TLR2 and TLR4 cell surface expression on monocytes from older adults (age 60–85) with that from young adults (age 18–35) and found no independent effect of age on TLR2 and TLR4 levels and LPS-induced cytokine production [21]. Furthermore, animal studies evaluating TLR levels and signaling in macrophages of old mice have yielded inconsistent findings. A study performed in young and old mice found that aged mouse macrophages have lower TLR4 protein [44]. In contrast, others have not observed differences in TLR4 cell surface expression in macrophages from aged versus young mice [43]. 

 In this study, we did not observe differences in JNK phosphorylation between groups. In contrast, studies in mice suggest that JNK plays an important role in the pathogenesis of obesity and insulin resistance [45]. To the best of our knowledge, most of the human studies supporting the role of JNK in insulin resistance have examined adipose tissue [46–48]. Moreover, our finding is in line with that of a previous study showing that JNK activity in PMNC was not associated with impaired insulin action [46], whereas increased JNK activity in adipose tissue directly correlated with measurements of insulin resistance [46].

 Studies of NF*κ*B activity in inflammatory cells from insulin-resistant subjects have yielded contrasting results. In line with a study by Patel et al., we did not observe differences in NF*κ*B activity in PMNC in insulin-resistant subjects [49]. In contrast, Ghanim et al. and Dasu et al. reported increased NF*κ*B activity in PMNC from obese individuals and monocytes (a subpopulation of PMNC) from type 2 diabetic subjects, respectively [17, 50]. Studies carried out in larger cohorts of subjects might help to clarify whether NF*κ*B signaling is up regulated in PMNC from obese and type 2 diabetic individuals and whether this alteration is specific to monocytes. 

## 5. Conclusions

 In summary, acute aerobic exercise improved insulin sensitivity and cardiovascular fitness in insulin-resistant subjects. However, exercise was not associated with changes in TLR4 protein content and ERK signaling in immune cells. It remains to be determined whether longer training protocols or different modes of exercise (i.e., resistance training or combination of aerobic and resistance training, as well as moderate- versus high-intensity exercise program) affect TLR4 expression and ERK signaling in immune cells of insulin-resistant individuals. 

## Figures and Tables

**Table 1:** Subject characteristics, metabolic parameters, and inflammatory markers pre and post exercise training.

	Lean		Obese		T2DM	
	*n* = 17		*n* = 8		*n* = 11	
	Pre	Post	Pre	Post	Pre	Post
Subject characteristics						
Age (years)	39 ± 2.0	—	40 ± 3.0	—	50 ± 3.0^∗†^	—
BMI (kg/m^2^)	25 ± 0.6	25 ± 0.7	31 ± 1.1*	31 ± 1.2^‡^	34 ± 0.7*	34 ± 0.5^‡^
Weight (kg)	66 ± 2.0	66 ± 2.3	81 ± 2.8*	82 ± 1.2^‡^	88 ± 3.1*	90 ± 2.7^‡^
Metabolic parameters						
NEFA AUC during OGTT (*μ*mol/L·2 h)	31 ± 2.2	—	40 ± 5.0	—	60 ± 5.6^∗†^	—
Fasting NEFA during OGTT (*μ*mol/L)	0.51 ± 0.1	—	0.63 ± 0.1	—	0.70 ± 0.0*	—
HbA_1c_ (%)	5.1 ± 0.1	—	5.0 ± 0.1	—	7.6 ± 0.8^∗†^	—
Fasting insulin (uIU/mL)	2.7 ± 0.5	2.2 ± 0.5	16 ± 6.3*	15 ± 3.6^‡^	9.5 ± 1.4*	9.9 ± 1.2^‡^
Fasting glucose (mg/dL)	93 ± 2	92 ± 2	95 ± 1	97 ± 1	132 ± 13^∗†^	139 ± 14^‡#^
Inflammatory markers						
hs-CRP (*μ*g/mL)	2.56 ± 1.0	2.35 ± 0.7	6.63 ± 3.14	5.36 ± 1.8	4.29 ± 0.8*	4.16 ± 1.0
Endothelin-1 (pg/mL)	2.43 ± 0.2	2.83 ± 0.3	3.42 ± 0.3	2.65 ± 0.2	4.53 ± 0.3*	4.38 ± 0.1
sICAM-1 (ng/mL)	210 ± 17	197 ± 11	172 ± 18	179 ± 17	271 ± 13*	263 ± 12^‡^

Data are mean ± SE; *P* < 0.05; *versus lean (preexercise); ^†^versus obese (preexercise); ^‡^versus lean (postexercise); ^#^versus obese (postexercise).

## References

[1] Pedersen BK (2006). The anti-inflammatory effect of exercise: its role in diabetes and cardiovascular disease control. *Essays in Biochemistry*.

[2] Duncan GE, Perri MG, Theriaque DW, Hutson AD, Eckel RH, Stacpoole PW (2003). Exercise training, without weight loss, increases insulin sensitivity and postheparin plasma lipase activity in previously sedentary adults. *Diabetes Care*.

[3] Green DJ, Maiorana A, O’Driscoll G, Taylor R (2004). Effect of exercise training on endothelium-derived nitric oxide function in humans. *Journal of Physiology*.

[4] Simpson RJ, McFarlin BK, McSporran C, Spielmann G, Hartaigh BO, Guy K (2009). Toll-like receptor expression on classic and pro-inflammatory blood monocytes after acute exercise in humans. *Brain, Behavior, and Immunity*.

[5] Steensberg A, Toft AD, Bruunsgaard H, Sandmand M, Halkjær-Kristensen J, Pedersen BK (2001). Strenuous exercise decreases the percentage of type 1 T cells in the circulation. *Journal of Applied Physiology*.

[6] Smith JA, Gray AB, Pyne DB, Baker MS, Telford RD, Weidemann MJ (1996). Moderate exercise triggers both priming and activation of neutrophil subpopulations. *American Journal of Physiology*.

[7] Balducci S, Zanuso S, Nicolucci A (2010). Anti-inflammatory effect of exercise training in subjects with type 2 diabetes and the metabolic syndrome is dependent on exercise modalities and independent of weight loss. *Nutrition, Metabolism and Cardiovascular Diseases*.

[8] Hevener AL, Olefsky JM, Reichart D (2007). Macrophage PPAR*γ* is required for normal skeletal muscle and hepatic insulin sensitivity and full antidiabetic effects of thiazolidinediones. *Journal of Clinical Investigation*.

[9] Feuerer M, Herrero L, Cipolletta D (2009). Lean, but not obese, fat is enriched for a unique population of regulatory T cells that affect metabolic parameters. *Nature Medicine*.

[10] Libby P (2002). Inflammation in atherosclerosis. *Nature*.

[11] Calles-Escandon J, Cipolla M (2001). Diabetes and endothelial dysfunction: a clinical perspective. *Endocrine Reviews*.

[12] Bobryshev YV (2006). Monocyte recruitment and foam cell formation in atherosclerosis. *Micron*.

[13] Reyna SM, Ghosh S, Tantiwong P (2008). Elevated Toll-like receptor 4 expression and signaling in muscle from insulin-resistant subjects. *Diabetes*.

[14] Tsukumo DML, Carvalho-Filho MA, Carvalheira JBC (2007). Loss-of-function mutation in Toll-like receptor 4 prevents diet-induced obesity and insulin resistance. *Diabetes*.

[15] Caricilli AM, Nascimento PH, Pauli JR (2008). Inhibition of Toll-like receptor 2 expression improves insulin sensitivity and signaling in muscle and white adipose tissue of mice fed a high-fat diet. *Journal of Endocrinology*.

[16] Mullick AE, Tobias PS, Curtiss LK (2006). Toll-like receptors and atherosclerosis: key contributors in disease and health?. *Immunologic Research*.

[17] Dasu MR, Devaraj S, Park S, Jialal I (2010). Increased Toll-Like Receptor (TLR) activation and TLR ligands in recently diagnosed type 2 diabetic subjects. *Diabetes Care*.

[18] Hasu M, Thabet M, Tam N, Whitman SC (2011). Specific loss of Toll-like receptor 2 on bone marrow derived cells decreases atherosclerosis in LDL receptor null mice. *Canadian Journal of Physiology and Pharmacology*.

[19] Michelsen KS, Wong MH, Shah PK (2004). Lack of Toll-like receptor 4 or myeloid differentiation factor 88 reduces atherosclerosis and alters plaque phenotype in mice deficient in apolipoprotein E. *Proceedings of the National Academy of Sciences of the United States of America*.

[20] McFarlin BK, Flynn MG, Campbell WW, Stewart LK, Timmerman KL (2004). TLR4 is lower in resistance-trained older women and related to inflammatory cytokines. *Medicine and Science in Sports and Exercise*.

[21] Stewart LK, Flynn MG, Campbell WW (2005). Influence of exercise training and age on CD14+ cell-surface expression of Toll-like receptor 2 and 4. *Brain, Behavior, and Immunity*.

[22] Nguyen MTA, Favelyukis S, Nguyen AK (2007). A subpopulation of macrophages infiltrates hypertrophic adipose tissue and is activated by free fatty acids via Toll-like receptors 2 and 4 and JNK-dependent pathways. *Journal of Biological Chemistry*.

[23] Varma V, Yao-Borengasser A, Rasouli N (2009). Muscle inflammatory response and insulin resistance: synergistic interaction between macrophages and fatty acids leads to impaired insulin action. *American Journal of Physiology—Endocrinology and Metabolism*.

[24] Karlmark KR, Wasmuth HE, Trautwein C, Tacke F (2008). Chemokine-directed immune cell infiltration in acute and chronic liver disease. *Expert Review of Gastroenterology and Hepatology*.

[25] Sriwijitkamol A, Coletta DK, Wajcberg E (2007). Effect of acute exercise on AMPK signaling in skeletal muscle of subjects with type 2 diabetes: a time-course and dose-response study. *Diabetes*.

[26] DeFronzo RA, Tobin JD, Andres R (1979). Glucose clamp technique: a method for quantifying insulin secretion and resistance. *The American Journal of Physiology*.

[27] Frøsig C, Sajan MP, Maarbjerg SJ (2007). Exercise improves phosphatidylinositol-3,4,5-trisphosphate responsiveness of atypical protein kinase C and interacts with insulin signalling to peptide elongation in human skeletal muscle. *Journal of Physiology*.

[28] Bouzakri K, Karlsson HKR, Vestergaard H, Madsbad S, Christiansen E, Zierath JR (2006). IRS-1 serine phosphorylation and insulin resistance in skeletal muscle from pancreas transplant recipients. *Diabetes*.

[29] Cipolletta D, Feuerer M, Li A (2012). PPAR-gamma is a major driver of the accumulation and phenotype of adipose tissue Treg cells. *Nature*.

[30] Oliveira M, Gleeson M (2010). The influence of prolonged cycling on monocyte Toll-like receptor 2 and 4 expression in healthy men. *European Journal of Applied Physiology*.

[31] Timmerman KL, Flynn MG, Coen PM, Markofski MM, Pence BD (2008). Exercise training-induced lowering of inflammatory (CD14+CD16+) monocytes: a role in the anti-inflammatory influence of exercise?. *Journal of Leukocyte Biology*.

[32] Häkkinen T, Karkola K, Ylä-Herttuala S (2000). Macrophages, smooth muscle cells, endothelial cells, and T-cells express CD40 and CD40L in fatty streaks and more advanced human atherosclerotic lesions: colocalization with epitopes of oxidized low-density lipoprotein, scavenger receptor, and CD16 (Fc*γ*RIII). *Virchows Archiv*.

[33] Devaraj S, Dasu MR, Rockwood J, Winter W, Griffen SC, Jialal I (2008). Increased Toll-like receptor (TLR) 2 and TLR4 expression in monocytes from patients with type 1 diabetes: further evidence of a proinflammatory state. *Journal of Clinical Endocrinology and Metabolism*.

[34] Saberi M, Woods NB, de Luca C (2009). Hematopoietic cell-specific deletion of Toll-like receptor 4 ameliorates hepatic and adipose tissue insulin resistance in high-fat-fed mice. *Cell Metabolism*.

[35] Jager J, Corcelle V, Grémeaux T (2011). Deficiency in the extracellular signal-regulated kinase 1 (ERK1) protects leptin-deficient mice from insulin resistance without affecting obesity. *Diabetologia*.

[36] Yao Y, Li W, Wu J (2003). Extracellular signal-regulated kinase 2 is necessary for mesoderm differentiation. *Proceedings of the National Academy of Sciences of the United States of America*.

[37] Haffner SM, Lehto S, Ronnemaa T, Pyörälä K, Laakso M (1998). Mortality from coronary heart disease in subjects with type 2 diabetes and in nondiabetic subjects with and without prior myocardial infarction. *New England Journal of Medicine*.

[38] Coenen KR, Gruen ML, Lee-Young RS, Puglisi MJ, Wasserman DH, Hasty AH (2009). Impact of macrophage Toll-like receptor 4 deficiency on macrophage infiltration into adipose tissue and the artery wall in mice. *Diabetologia*.

[39] Hu X, Moscinski LC, Valkov NI, Fisher AB, Hill BJ, Zuckerman KS (2000). Prolonged activation of the mitogen-activated protein kinase pathway is required for macrophage-like differentiation of a human myeloid leukemic cell line. *Cell Growth and Differentiation*.

[40] Ashida N, Arai H, Yamasaki M, Kita T (2001). Distinct signaling pathways for MCP-1-dependent integrin activation and chemotaxis. *Journal of Biological Chemistry*.

[41] Wang L, Li D, Yang K, Hu Y, Zeng Q (2008). Toll-like receptor-4 and mitogen-activated protein kinase signal system are involved in activation of dendritic cells in patients with acute coronary syndrome. *Immunology*.

[42] Esposito G, Perrino C, Schiattarella GG (2010). Induction of mitogen-activated protein kinases is proportional to the amount of pressure overload. *Hypertension*.

[43] Boehmer ED, Goral J, Faunce DE, Kovacs EJ (2004). Age-dependent decrease in Toll-like receptor 4-mediated proinflammatory cytokine production and mitogen-activated protein kinase expression. *Journal of Leukocyte Biology*.

[44] Renshaw M, Rockwell J, Engleman C, Gewirtz A, Katz J, Sambhara S (2002). Cutting edge: impaired Toll-like receptor expression and function in aging. *Journal of Immunology*.

[45] Hirosumi J, Tuncman G, Chang L (2002). A central, role for JNK in obesity and insulin resistance. *Nature*.

[46] Sourris KC, Lyons JG, de Courten MPJ (2009). c-Jun NH2-terminal kinase activity in subcutaneous adipose tissue but not nuclear factor-*κ*B activity in peripheral blood mononuclear cells is an independent determinant of insulin resistance in healthy individuals. *Diabetes*.

[47] Blüher M, Bashan N, Shai I (2009). Activated Ask1-MKK4-p38MAPK/JNK stress signaling pathway in human omental fat tissue may link macrophage infiltration to whole-body insulin sensitivity. *Journal of Clinical Endocrinology and Metabolism*.

[48] Boden G, Duan X, Homko C (2008). Increase in endoplasmic reticulum stress-related proteins and genes in adipose tissue of obese, insulin-resistant individuals. *Diabetes*.

[49] Patel C, Ghanim H, Ravishankar S (2007). Prolonged reactive oxygen species generation and nuclear factor-*κ*B activation after a high-fat, high-carbohydrate meal in the obese. *Journal of Clinical Endocrinology and Metabolism*.

[50] Ghanim H, Aljada A, Hofmeyer D, Syed T, Mohanty P, Dandona P (2004). Circulating mononuclear cells in the obese are in a proinflammatory state. *Circulation*.

